# Riverine Health Assessment Using Coordinated Development Degree Model Based on Natural and Social Functions in the Lhasa River, China

**DOI:** 10.3390/ijerph19127182

**Published:** 2022-06-11

**Authors:** Junhong Chen, Yanjun Kong, Yadong Mei

**Affiliations:** 1 Guangxi Key Laboratory of Environmental Pollution Control Theory and Technology, Guilin University of Technology, Guilin 541004, China; 2 Collaborative Innovation Center for Water Pollution Control and Water Safety in Karst Area, Guilin University of Technology, Guilin 541004, China; 3 Changjiang Survey, Planning, Design and Research Co., Ltd., Wuhan 430011, China; kongyanjun@whu.edu.cn; 4 State Key Laboratory of Water Resources and Hydropower Engineering Science, Wuhan University, Wuhan 430072, China; ydmei@whu.edu.cn

**Keywords:** coordinated development degree model, riverine health assessment, index system, Lhasa River

## Abstract

Rivers provide a variety of ecosystem services to humans. However, human interference significantly impairs the rivers’ functions and poses a threat to river health. To increase the understanding of riverine health in Tibet, China from 2011 to 2014, this study used the Lhasa River as a case study and established a multiple indicator system incorporating both natural and social functions of the river. Weights of riverine health indicators were calculated using the entropy method. Moreover, to evaluate the coordination and development of natural and social functions, a coordinated development degree model was developed. The results showed that the entropy weights of natural and social functions in the target layer were 0.67 and 0.33, respectively. Natural functions, social functions, and riverine state index all decreased from upstream to downstream, and marked as “good” during the entire study period. In 2012, the coordinated development degree improved from previously “moderately coordinated” to “highly coordinated”. Furthermore, the development of natural and social functions was synchronized throughout the study period. Further analysis revealed that the construction of hydraulic projects had a significant effect on the hydrological regime, resulting in an increase in social functions of the river. Therefore, the coordinated development degree model is shown to provide new insight into assessing riverine health in terms of both natural and social functions.

## 1. Introduction

Rivers provide the material foundation and guarantee for survival and economic development. Meanwhile, they also play a critical role in maintaining the biosphere, water cycle, materials, and energy balance [1]. With rapid population growth and economic development, rivers are becoming less capable of meeting societal needs, thus the pressure placed on riverine ecosystems is increasing. More than 98,000 reservoirs and dams have been launched on the Chinese rivers in the last 70 years to improve their functionality and obtain additional ecological resources [2]. Humanity has benefited greatly from river development and utilization. For example, reservoir construction has increased the sustainability of water supplies, hydraulic diversion projects have reduced flood-related losses, and hydroelectric power has improved energy supplies [3]. However, due to a lack of scientific guidance, the structure and function of riverine ecosystems have been harmed by overexploitation and utilization [4]. Reduced water resources, environmental degradation, soil erosion, and habitat destruction are all common occurrences throughout China, affecting not only human production and lives, but also regional economic and social development [5]. Long-term socioeconomic development can be achieved only by coordinating water resource exploitation and environmental protection [6]. Therefore, riverine ecosystem health assessments aid in sustainable development by improving knowledge regarding riverine health and change, allowing for scientific river management and ecological restoration [7].

The definition of riverine ecosystem health has not been unified since it was proposed. In accordance with Scrimgeour and Wicklum [8], healthy rivers are those that can maintain basic function, whereas Karr [9] used the balance, integrity, and adaptability of the riverine ecosystem as health evaluation criteria. Furthermore, Gao et al. [10] defined a healthy river as one that has good ecology, meets human needs, and achieves sustainable and efficient water resource use. With a better understanding of the riverine ecosystem, the concept has expanded beyond ecological integrity to include social, economic, and cultural factors [11]. Natural functions, such as hydrological, physical, chemical, and biological characteristics can provide a more accurate representation of a river’s health [12,13]. Natural rivers acquire additional social characteristics as a result of continuous changes caused by human activity [14]. Rivers must not only maintain stability in an ever-changing environment, but also perform critical functions, such as flood control, irrigation, water supply, transportation, recreation, fisheries, and other social functions [15]. Therefore, a healthy river should not only maintain natural integrity, but also social integrity [16].

Riverine health assessments, as a critical component of ecosystem health assessments, reflect river health status and trends to promote a sustainable development of the human–water relationship [17]. In general, riverine health assessment focuses on the development of index systems from four perspectives: Hydrology, habitat, biology, and water quality. However, there is no unified standard or method for assessing riverine health that is based on the geographical characteristics, climatic conditions, economic development, and social needs of the assessed objects. Currently, methods for assessing riverine health include the TOPSIS model [18], fuzzy matter element analysis [19], artificial neural network [20], and projection pursuit regression [21]. Riverine health assessment is viewed as a decision-making process fraught with uncertainty due to the presence of multiple indicators with varying characteristics [16]. These evaluation methods produce a comprehensive index reflecting overall health conditions. However, it is devoid of natural and social functions analysis and does not reveal the interactions and comparative relationships between the subsystems.

Therefore, coordinated development degree model can reflect both the synergy level among system elements and the level of comprehensive development of the overall system [22,23]. Coordinated development focuses on enhancing the integrity and comprehensiveness of systems, which is necessary for sustainable development [24]. Furthermore, the coordinated development degree model is widely used in socioeconomics. Wang et al. [25] and Xing et al. [26] used the link between energy, economy, resources, and environment as the entry points to investigate the socioeconomic systems’ coordinated development patterns. Moreover, few studies have been conducted on riverine health assessments, particularly on the correlation and interaction between the factors influencing riverine health. By incorporating the coordinated development degree model into the riverine health assessment, not only can the overall development level be determined, but also the synergy level between the internal influencing factors, thereby providing a foundation for scientific riverine ecosystem management.

The Lhasa River Basin, located in the vicinity of the Qinghai-Tibet Plateau, has the most developed economy and densest population in the Tibet Autonomous Region. To ensure the coordinated development of the social economy and natural environment in the Lhasa River Basin, one of the critical measures is the rational and effective development of the Lhasa River. Urbanization and population growth, combined with other factors, such as extremely uneven runoff distribution and water pollution accidents, have potentially threatened the Lhasa River’s health and environmental protection in recent years [27]. Additionally, the Lhasa River Basin is an ecologically fragile region. There are several environmental issues, including invasive alien species, non-point agricultural source pollution, and the decline of indigenous fish species [28]. Moreover, increased runoff during the dry season and changes in riparian vegetation as a result of natural variability and human activity have an influence on riverine health [28,29]. Therefore, to comprehend the health status and main issues of the Lhasa River, a method for assessing riverine health is required.

The main goal of this paper was to establish a comprehensive multiple indicator system that encompassed both natural and social functions, as well as to develop a coordinated development degree model that accounted for both natural and social functions. Finally, the objective was to apply this model to the Lhasa River Basin to evaluate the spatial and temporal variability of riverine health and to analyze the major factors affecting the evaluation results.

## 2. Materials and Methods

### 2.1. Study Area

**Figure 1:**
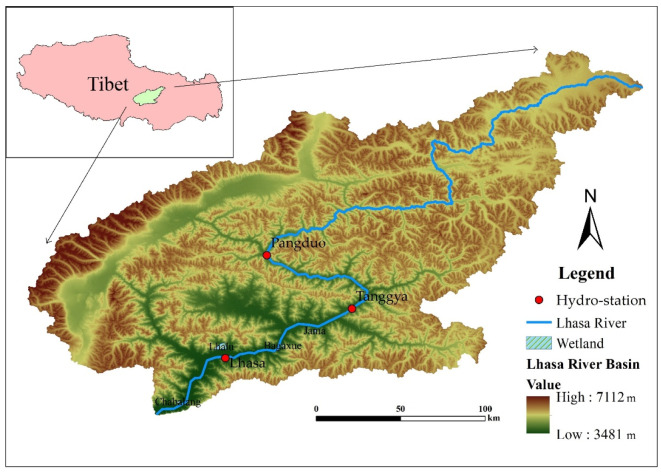
The location of the Lhasa River.

The Lhasa River (Figure 1) is one of five tributaries at the middle course of the Yarlung Tsangpo River. The main stream is 551 km long and has an average gradient of 0.29%, extending in an “S” shape from northeast to southwest. The stream originates in a flat wetland at an elevation of 5283 m and flows into the Yarlung Tsangpo River, losing approximately 1700 m of elevation along the way. It is primarily fed by precipitation, snowmelt, and groundwater, which account for 46%, 26%, and 28%, respectively, of total runoff. The Lhasa River Basin covers an area of 32,471 km^2^ (29°20′~31°15′ N, 90°05′~93°20′ E), accounting for 13.5% of the area of the Yarlung Tsangpo River Basin, and has an average elevation of approximately 4500 m. Due to the river’s flat terrain and abundant soil resources, the middle and lower reaches of the Lhasa River have the densest population and most cultivated areas in Tibet. The Lhasa River’s average annual runoff is 1.097 × 10^10^ m^3^, indicating a significant potential for water and hydropower resources. The Pangduo and Zhikong hydropower stations have extraordinary regulating effects on the river’s hydrological processes. The Lhasa River flows through the central part of the Qinghai-Tibet Plateau, which has a semi-arid climate within a temperate plateau. The basin’s annual average temperature is 5.3 °C and the average annual precipitation is 400–500 mm [16]. Influenced by warm and humid air coming from the Indian Ocean, precipitation primarily occurs during summer and decreases from upstream to downstream. The precipitation intensity is low, while evaporation is high. The basin contains numerous plateau wetlands, such as the Lhalu, Jiangxia, Bagaxue, Jama, and Chabalang wetlands. These wetlands are critical for the Lhasa River Basin’s water cycle, water quality protection, and biodiversity.

### 2.2. Index System for Riverine Health Assessment

The development of an index system is the cornerstone of riverine health assessment. The scientific nature, rationality, and efficacy of the index system directly affect the credibility of the results of riverine health assessments. The index system is constructed in accordance with the principles advanced by Singh and Saxena [7]. The most frequently used index systems for evaluating riverine health are mainly based on ecosystem integrity, thermodynamics, and the pressure, state, and response framework [30,31]. Due to the fact that each riverine system is unique in terms of location, scale, environment, water supply, runoff, sediment, and other factors, the structure and function of each river system are quite distinct. Therefore, the corresponding index system for riverine health assessment should be specifically tailored to these characteristics.

**Figure 2:**
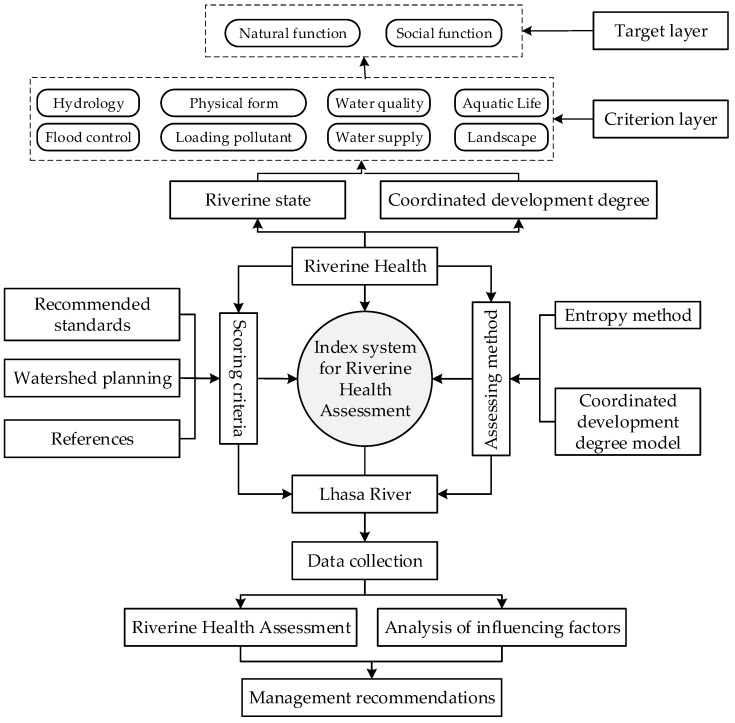
The overall framework of riverine health assessment index system.

On the basis of the abovementioned principles, this study developed an index system for assessing riverine health of the Lhasa River. First, a comprehensive literature review was conducted to determine the proper indicators and quantification methods, as well as the index weight calculation. Second, the environmental and social characteristics of the Lhasa River Basin, such as topography, climate, economy, and ecosystem services were analyzed through field investigation. Finally, after incorporating the natural and social characteristics of the Lhasa River Basin, a three-layer (A: Target layer, B: Criterion layer, C: Index layer) index system for assessing riverine health was developed. The index system is illustrated in Table 1. The overall framework of riverine health assessment index system on the Lhasa River is shown in Figure 2.

The target of the index system was riverine health, which could be defined in terms of natural and social functions. Natural functions were classified into four categories based on the characteristics of the Lhasa River. These categories include hydrology, physical form, water quality, and aquatic life. Hydrology criteria consisted of flow deviation (C1) and ecological flow satisfaction (C2), both of which can be used to directly reflect changes in water quantity and interact with aquatic ecosystems [32]. C1 denotes the difference between measured and natural runoff, whereas C2 denotes the minimum runoff required to maintain fish and aquatic plant survival and population structure. The physical form of a river was determined by its riparian status (C3), river connectivity (C4), and wetland retention (C5), all of which may reflect human activities that alter river morphology [33]. C3 is a broad index that incorporated riverbank stability, fractional vegetation cover, and riparian disturbance. Riverbank stability was dependent on slope, altitude, materials composition, and degree of erosion. Moreover, fractional vegetation cover refers to the percentage of the surface vegetation area in the study area [34]. Additionally, riparian disturbance refers to the adverse effects of human activities on riparian systems, which include home, pipeline, and railroad construction, waste disposal, mining, agricultural cultivation, and farming. Three indicators were used to assess water quality: Dissolved oxygen (C6), nutrients, and toxicity. These factors can reflect the overall water quality of the Lhasa River [35]. Furthermore, minimum scores for organic pollutants, such as COD_Mn_, BOD_5_, COD, and NH_3_–N composed the nutrient concentration index (C7). Integrity index (C8) measures water pollution caused by heavy metals and compounds with high biological toxicity, such as mercury, cadmium, chromium, lead, and arsenic. The Shannon–Wiener biodiversity index (C9) and fish index (C10) were used to represent the aquatic life. These indices refer to the stabilization and regulation of aquatic ecosystems via biological communities [36]. On the other hand, social services were encapsulated by the materials and functions provided by rivers for human well-being and development, including flood control, pollution response, power generation, shipping, water supply, irrigation, landscape, leisure, and culture [37]. The flood control index (C11) indicates the degree of protection from flooding. The water function zone index (C12) was calculated as the ratio of water function regions with water quality that meets the standard (GB3838-2002 and GB/T50594-2010) to all water function regions. The index of water resource utilization (C13) indicates the proportion of water supply to total water resources in the river basin. The index of water supply (C14) is a ratio of total water supply to total water demand that indicated the river’s ability to meet water demand for economic development in the surrounding areas. The index of hydropower development (C15) refers to the ratio of developed installed capacity and potential hydropower resources. The index of public satisfaction (C16) measured public satisfaction with river landscape, as well as its aesthetic value. It evaluates the comfort level of human senses brought by water landscape, leisure, and cultural places of the river by means of questionnaire survey. The supplementary material contains a detailed description and calculation method for each index (Table S1).

The original data obtained in this study had a variety of dimensions. To improve the comparability of different evaluation results, it was necessary to standardize the quantification and evaluation criteria for riverine health as much as possible. In this study, indicators were rescaled to a score between 0 and 1. When the index value was equal to or greater than the optimal value, the score was 1, whereas when the index value was equal to or less than the lowest value, the score was 0 [38]. When the value was between the best and the worst thresholds, the index score was calculated using linear interpolation, as shown in the left part of Equation (1). A negative index score was calculated in the inverse manner, as can be seen in the right part of Equation (1):

(1) $$s_{i}=\left\{ \begin{array}{c} \;\;\;\;0\;\;\;\;\;\;x_{i}\leq a_{i}\\ 0.2\left(x_{i}-a_{i}\right)/\left(b_{i}-a_{i}\right)\;\;\;a_{i}<x_{i}\leq b_{i}\\ 0.2+0.1\left(x_{i}-b_{i}\right)/\left(c_{i}-b_{i}\right)\;b_{i}<x_{i}\leq c_{i}\\ 0.3+0.1\left(x_{i}-c_{i}\right)/\left(d_{i}-c_{i}\right)\;c_{i}<x_{i}\leq d_{i}\\ 0.4+0.2\left(x_{i}-d_{i}\right)/\left(e_{i}-d_{i}\right)\;d_{i}<x_{i}\leq e_{i}\;\\ 0.6+0.2\left(x_{i}-e_{i}\right)/\left(h_{i}-e_{i}\right)\;e_{i}<x_{i}\leq h_{i}\\ 0.8+0.2\left(x_{i}-h_{i}\right)/\left(k_{i}-h_{i}\right)\;h_{i}<x_{i}\leq k_{i}\\ \;\;\;\;\;1\;\;\;\;\;\;\;k_{i}<x_{i} \end{array}\right.;\;s_{i}=\left\{ \begin{array}{c} \;\;\;\;1\;\;\;\;\;\;\;\;x_{i}\leq k_{i}\\ 0.8+0.2\left(h_{i}-x_{i}\right)/\left(h_{i}-k_{i}\right)\;\;\;\;k_{i}<x_{i}\leq h_{i}\\ 0.6+0.2\left(e_{i}-x_{i}\right)/\left(e_{i}-h_{i}\right)\;\;\;\;h_{i}<x_{i}\leq e_{i}\\ 0.4+0.2\left(d_{i}-x_{i}\right)/\left(d_{i}-e_{d}\right)\;\;\;\;e_{i}<x_{i}\leq d_{i}\\ 0.3+0.1\left(c_{i}-x_{i}\right)/\left(c_{i}-d_{i}\right)\;\;\;\;d_{i}<x_{i}\leq c_{i}\;\\ 0.2+0.1\left(b_{i}-x_{i}\right)/\left(b_{i}-c_{i}\right)\;\;\;\;c_{i}<x_{i}\leq b_{i}\;\\ 0.2\left(a_{i}-x_{i}\right)/\left(a_{i}-b_{i}\right)\;\;\;\;\;\;\;b_{i}<x_{i}\leq a_{i}\;\\ \;\;\;\;0\;\;\;\;\;\;\;\;a_{i}<x_{i} \end{array}\right.$$

where *s_i_* represents index score *i*, *s_i_* ∈ [0, 1]; *x_i_* is the value of index *i*; and *a_i_*, *b_i_*, *c_i_*, *d_i_*, *e_i_*, *h_i_*, and *k_i_* signify the threshold values of index *i*.

### 2.3. Data Collection

This research used three hydrological monitoring stations in Pangduo, Tanggya, and Lhasa, to determine the riverine health status and the coordinated development degree in the Lhasa River’s upstream, midstream, and downstream segments. Daily discharge measurements were obtained from monitoring stations. Data on water quality were collected from the Tibet Autonomous Region Water Environment Monitoring Center. In terms of biological data, they were obtained from the historical information and field investigation on fishery resources in the Lhasa River Basin [39]. Data on the water function zone, water supply, water utilization, and water drainage were obtained from Tibet Water Resources Bulletin. Additionally, data on the public satisfaction were obtained by the questionnaire. Detailed information are shown in Table 1.

### 2.4. The Scoring Criteria of Riverine Health Indicators

Due to differences in topography, climate, and economy, the objectives of riverine health assessments vary significantly, making it difficult to standardize the indicators and scoring criteria. The critical threshold and expert consultation approaches were used in this paper to determine the scoring criteria for riverine health indicators based on the characteristics of the watershed environment. The critical threshold method considers the river ecosystem in its natural state as the ideal state and factors in human interference and social demand [40]. The original state without interference is the optimal value, with a score of 1.0. Then, as the degree of disturbance increases, the score reduces. Due to the inability of some indicators to determine the non-interference state or natural state, the indicator health scores were compared with the existing standards [41] and the River and Lake Health Assessment Guide [42]. For instance, C12 was calculated by determining whether the river’s water quality complies with the standard for water function zone. The specific scoring criteria for riverine health indicators are presented in Table 2.

### 2.5. Weight Estimation

In the field of information theory, entropy is frequently used to quantify the degree of information chaos [18]. This research used the information entropy weight method to reflect the differences between indicators in the different systems. The lower the entropy, the greater the entropy weight and the development of index [43]. The equation was used as follows:

(2) $$w_{i}=\frac{1-S}{m-\sum_{j=1}^{m}S}$$

(3) $$S=-\frac{1}{\ln n}\sum_{i=1}^{n}\frac{s_{ij}}{s^{*}}\ln\frac{s_{ij}}{s^{*}},s^{*}=\sum_{i=1}^{n}s_{ij}$$

where *w_i_* is the entropy weight; *m* represents the number of indicators; *S* signifies the information entropy of index *j*; and s^*^ is the total score of index *j*. The results of index weight are presented in the supplementary material (Table S2).

### 2.6. Coordinated Development Degree Model

The coordinated development degree model is a systematic method used to quantitatively study the coordination relationship among several systems. In this paper, the coordinated development theory is used to evaluate the contradiction and harmony between the natural and social functions of a riverine ecosystem. The riverine health index is a quantitative indicator of a river’s health. Natural function index scores were denoted by *x*_1_, *x*_2_, …, *x_p_*, while social functions were expressed by *y*_1_, *y*_2_, …, *y_q_*. Then, for each river monitoring point, the natural functions *f*(*x*) and social functions *g*(*y*) were expressed as follows:

(4) $$f\left(x\right)=\sum_{i=1}^{p}w_{i}x_{i}$$

(5) $$g\left(y\right)=\sum_{j=1}^{q}w_{j}y_{j}$$

where *w_i_* and *w_j_* represent the weight of each natural and social function indicator, respectively. Furthermore, *p* and *q* signify the number of natural and social function indicators. In this paper, *p* and *q* are equal to 10 and 6, respectively.

The greater the value of natural functions *f*(*x*) and social functions *g*(*y*), and the lower the coefficient of deviation between *f*(*x*) and *g*(*y*), the healthier the riverine ecosystem. The synergy between the river’s natural and social functions can be measured by calculating the coordination degree of the functions:

(6) $$C=\sqrt{\left(1-C_{fg}^{2}\right)}$$

(7) $$C_{fg}=\sqrt{1-\frac{f\left(x\right)\cdot g\left(y\right)}{{\left(\frac{f\left(x\right)+g\left(y\right)}{2}\right)}^{2}}}$$

where *C* is the coordination degree between *f*(*x*) and *g*(*y*). *C_fg_* is the deviation coefficient between *f*(*x*) and *g*(*y*), which can reflect the coordination degree between the subsystems. *C* increases as *C_fg_* decreases, indicating the higher coordination between the subsystems.

A high coordination degree may reflect the similar rate of development of both natural and social functions in riverine ecosystems, but it is possible for both to be at a low level of development [44]. Therefore, it is difficult to accurately assess riverine health using only the coordination degree calculation. Coordinated development degree can be used to reflect the coordination and development status of natural and social functions concurrently. The formulas are as follows:

(8) $$T=W_{i}f\left(x\right)+W_{j}g\left(y\right)$$

(9) $$D=\sqrt{CT}$$

where *T* represents the composite evaluation index that reflects the health state of riverine ecosystems. *W_i_* and *W_j_* signify the integrated weights of natural and social functions, and they are calculated as $W_{i}=\sum_{i=1}^{p}w_{i}$, $W_{j}=\sum_{j=1}^{q}w_{j}$, respectively. Moreover, *D* is the coordinated development degree, used to evaluate the status of coordinative development of natural and social function subsystems.

To further assess the relative development status of natural and social functions in riverine ecosystems, this study introduced a calculation for the relative development degree of natural and social functions in the Lhasa River [43]. Its formula is as follows:

(10) $$E=\frac{f\left(x\right)}{g\left(y\right)}$$

The overall coordinated development degree can be obtained by the overall natural *F*(*x*) and social functions *G*(*y*):

(11) $$F\left(x\right)=\sum_{i=1}^{N}\frac{f{\left(x\right)}_{i}\cdot L_{i}}{L}$$

(12) $$G\left(y\right)=\sum_{j=1}^{N}\frac{g{\left(y\right)}_{j}\cdot L_{j}}{L}$$

where *f*(*x*)*_i_* and *g*(*y*)*_j_* represent the natural and social functions of each monitoring station, respectively; *L_i_* and *L_j_* signify the length of the river section observed by the monitoring point *i* and *j*, respectively; *L* represents the total river length; and *N* represents the number of riverine monitoring points. The overall coordination degree (*C_s_*), the overall composite evaluation index (*T_s_*), the overall coordinated development degree (*D_s_*), and the overall relative development degree (*E_s_*) were obtained based using Equations (6)–(10).

The evaluation criteria and classification standards serve as the relative scales for assessing the state of rivers and the coordination of their development. Both the riverine state index and the coordinated development degree were scaled from 0 to 1. When the values of riverine state index and coordinated development degree are equal to 1, the river ecosystem is in an optimal state of health and the subsystems are perfectly coordinated. On the other hand, index values equal to 0 indicate that the river ecosystem is in the worst possible condition and that each subsystem is completely uncoordinated. The ratio of natural and social functions represents the relative development degree. If it is less than 0.8, the natural functions are out of sync with social functions or social development has caused severe harm to the riverine ecosystem. When the relative development degree exceeds 1.2, social functions lag behind natural functions, indicating that the river ecosystem still has significant potential for social development. If the relative development degree is between 0.8 and 1.2, natural and social functions develop synchronously.

Table 3 summarizes the evaluation criteria and classification standards for the riverine state index, coordinated development degree, and relative development degree in accordance with Environmental Quality Standard for Surface Water [41] and the previous study [44]. The riverine state index was ranked by the uniformly distributed five grades with uniform distribution of critical, poor, medium, good, and excellent. Additionally, coordinated development degree was marked by the uniformly distributed five grades, including severely uncoordinated, moderately uncoordinated, barely coordinated, moderately coordinated, and highly coordinated development. On the other hand, the relative development degree was divided into three grades, including slow development of the natural functions, synchronized development, and slow development of the social functions.

## 3. Results

### 3.1. The Annual Variability of the Individual Indexes

**Figure 3:**
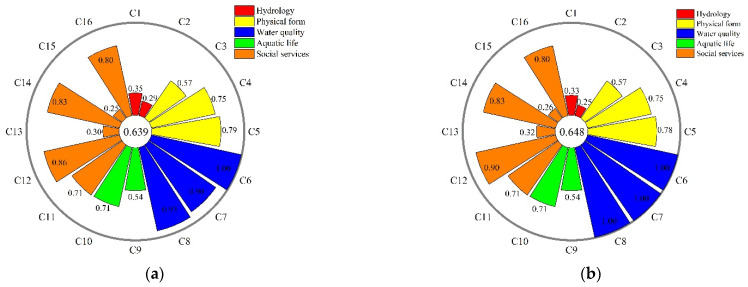

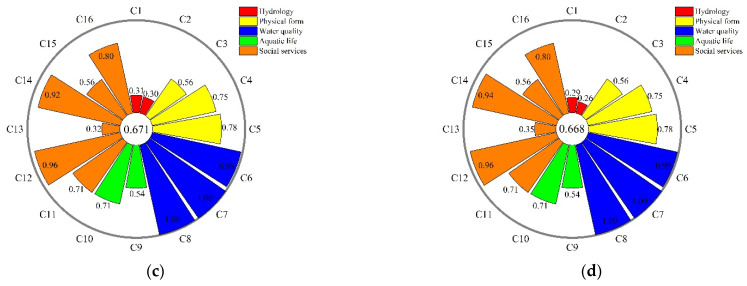
The annual variability of each index in the Lhasa River health assessments in (**a**) 2011; (**b**) 2012; (**c**) 2013; and (**d**) 2014.

The scores of different indexes varied greatly in the Lhasa River during the study period. Figure 3 illustrates the scores for each index from the period of 2011 to 2014. In terms of the indicators of hydrology, both the flow deviation index (C1) and the ecological flow satisfaction (C2) were in the poor state. The deviation between the measured runoff and natural runoff was primarily explained by the exploitation of basin water resources. It is important to note that C1 scores gradually decreased from upstream to downstream measuring points, as well as when comparing data from 2011 to 2014. This could indicate that human activities, such as water exploitation and dam construction have altered the natural flow of the Lhasa River. Furthermore, C2 scores for 2011 and 2013 were 0.29 and 0.30, respectively, which were higher than those measured in 2012 and 2014. Additionally, C2 score measured in Tanggya was lower than in Lhasa and Pangduo, indicating spatial variations in the index of natural functions. In general, low C1 and C2 scores, particularly in Tanggya and Lhasa, were taken as the primary reason for the decline in the natural function index of the area.

In terms of the physical indicator scores, they remained constant. C3 scores obtained in 2011 and 2012 were slightly higher than those for 2013 and 2014. Human activities, such as sand mining and road construction near the Lhasa River’s midstream and downstream had degraded the natural state of the riparian zone, resulting in a decrease in upstream C3 compared with downstream. Moreover, the spatial variation of the natural function index was also affected. On the other hand, C5 was slightly higher in 2011 than from 2012 to 2014, indicating that the Lhasa River Basin’s natural wetland areas had not significantly changed. River connectivity (C4), which was used to measure impediments to fish migration, water flow, and nutrient transfer, had also remained constant.

During the study period, indicators of water quality criterion were graded as excellent. DO concentration (C6) score was greater than 0.98 all throughout the study. C7 and C8 had an average score of 0.90 and 0.93, respectively in 2011. Between 2012 and 2014, C7 and C8 values were below the grade Ⅰ limits set by the Environmental Quality Standards for Surface Water (GB3838-2002), indicating that the Lhasa River’s water quality was good. In terms of the indicators that signify the aquatic life, the Shannon–Wiener biodiversity index (C9) and fish losses index (C10) remained constant with the scores of 0.54 and 0.71. However, due to the difficult research conditions in the Lhasa River Basin, there was a lack of biological data. Therefore, this study calculated C9 and C10 using the data obtained by the Chinese Academy of Sciences and Shaanxi Institute of Fisheries, which did not accurately reflect annual change. This shortcoming directly impaired the accuracy of the assessment results [39].

For the indicators of social functions, the flood control index (C11) scores were considered. They remained at 0.71, which indicates that the majority of dykes and bank protection met the flood control requirements of the Lhasa River. C12 scores were graded as excellent and had gradually increased from 2011 to 2014, indicating that the water quality mostly meets the set standard. The water resources utilization index (C13) remained poor throughout the study. However, it is notable that it had gradually been increasing from 0.25 to 0.35 during the study period. As shown in Table 2, it is recognized that a reasonable water resource utility rate should be between 30% and 40% [42]. The Lhasa River’s main stream had a low overall water resource utilization, while there were no large-scale hydraulic projects in the upstream. The construction and operation of the Pangduo Project increased the irrigation water availability in the Pengbo Irrigation Area, indicating an increasing human influence on water quantity. In terms of the water supply index (C14), it was marked as excellent in 2011 and 2012, with the value of 0.83, while it increased to 0.92 in 2013 and 0.94 in 2014. Another factor that changed over time is the hydropower development index (C15). In 2011 and 2012, it was marked as poor with the scores of 0.25 and 0.26, but it rapidly jumped to 0.56 in 2013 and 2014. This radical change represents the improvement in social function of the river. Although hydropower resources continued to have significant development and utilization potential, the completion of the Pangduo Hydraulic Project in 2013 had greatly increased the water resource regulation capacity, as well as the power supply for the area [45]. Furthermore, the results indicate that between 2011 and 2014, the Lhasa River Basin had high C16 scores, which were classified as excellent.

### 3.2. The Spatial and Temporal Variability of Coordinated Development

**Figure 4:**
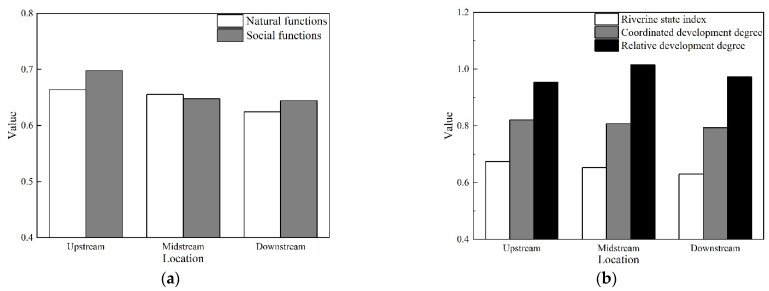
The overall results of the coordinated development degree assessment in the upstream, midstream, and downstream Lhasa River. The specific indicators include: (**a**) The natural and social functions; (**b**) the riverine state index, coordinated and relative development degree.

**Figure 5:**
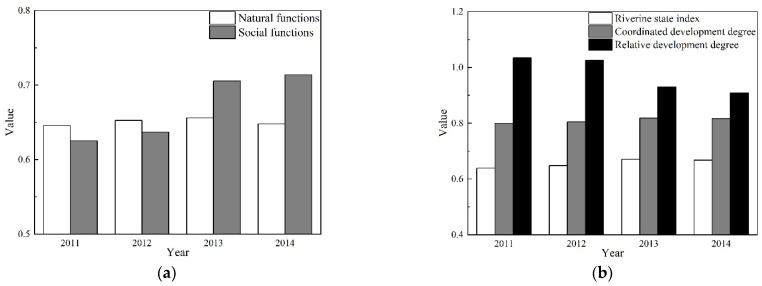
The overall results of the coordinated development degree assessment for the Lhasa River from 2011 to 2014. The specific indicators include: (**a**) Natural and social functions; (**b**) the riverine state index, coordinated and relative development degree.

The spatial and temporal variability of natural and social functions, riverine state index, coordinated development degree, and relative development degree in the Lhasa River were calculated using Equations (2)–(10). The calculations were based on the coordinated development degree model, while the results can be seen in Figure 4 and Figure 5. The health status of the Lhasa River was determined in accordance with the evaluation criteria and the classification standards are presented in Table 3.

The spatial variability of coordinated development degree assessment from the river’s upstream to downstream can be seen in Figure 4a,b. Natural function values of 0.664, 0.654, and 0.615 indicate a good status, while they gradually decreased from upstream to downstream. Social function results also decreased from upstream to downstream. Their status was marked as good. However, the values were greater than the natural function results. In terms of the relative development degree, the results had no clear spatial variability, while they all belonged to the criteria of the synchronized development. The respective values from upstream to downstream were 0.954, 1.015, and 0.973, respectively. Moreover, the riverine state index decreased from upstream to downstream and it had a “good” status all throughout, with values of 0.674, 0.653, and 0.630, respectively. Similarly, the coordinated development degree also decreased gradually from upstream to downstream, with its respective values as 0.821, 0.808, and 0.794, respectively.

Figure 5a,b illustrates the temporal variability of the assessed coordinated development degree measured on the Lhasa River from 2011 to 2014. The overall results for natural functions were all marked as good. This demonstrates that the Lhasa River’s natural environment remained constant throughout the research period. However, the overall results for social function for 2011 and 2012 were ranked as good, with values of 0.625 and 0.637, respectively, and then rapidly increased to 0.705 and 0.714 in 2013 and 2014, respectively. The overall relative development degree consistently indicated the synchronized development grade. Its values were 1.034 and 1.025 in 2011 and 2012, respectively, after which it decreased to 0.930 and 0.908 in 2013 and 2014, respectively, indicating that social function values surpassed the natural function ones. The riverine state index increased throughout the research period, although they were consistently classified as good, reaching the values of 0.639, 0.648, 0.671, and 0.668 for the years of 2011 to 2014, respectively. The coordinated development degree was classified as moderately coordinated development in 2011 and then as highly coordinated development from the years of 2012 to 2014, with the values of 0.805, 0.819, and 0.817, respectively.

## 4. Discussion

### 4.1. Natural Functions

Water resources in the study area are abundant, but also temporally heterogeneous. River discharge from May to October is concentrated and accounts for over 85% of the total amount of the annual discharge [28]. This may cause the lower scores of C1 and C2. The Lhasa River was not heavily exploited in terms of natural resources, and there were no large-scale hydraulic projects in the upstream. The Lhasa River’s natural flow pattern had been significantly altered by the construction of Pangduo and Zhikong water conservancy projects [45]. The main objectives of Pangduo are irrigation and energy generation. The average annual diversion flow of the Pangduo water conservancy project was measured as 2 × 10^9^ m^3^, which was mainly used to irrigate the farmland downstream. Similarly, the water from the Zhikong hydropower station can be used to irrigate the 5000 ha of farmland surrounding the area. Therefore, water storage, power generation, and irrigation of hydropower stations can all have an effect on the riverine hydrological regime. Pangduo and Zhikong water conservancy projects can store floodwater that is accumulated during the wet periods and respond to the environmental water demand in dry periods based on the dispatching schedules. However, discharge data from Pangduo, Tanggya, and Lhasa hydrological stations indicated that the downstream discharge decreases during drought and normal periods and increases during wet periods following the operation of Pangduo hydropower station, resulting in significant variation in riverine hydrological processes.

Additionally, climate change had a significant impact on the hydrological regimes of rivers. In accordance with Wu et al. [28] and Lin et al. [46], precipitation had decreased in the Lhasa River Basin since 2004, while temperature had continued to rise. Climate change directly affected the riverine hydrological regime. Decreased precipitation reduces water quantity in the river, while the influence of rising temperature on riverine hydrological regime is uncertain [47]. When the temperature rises, glacier melting leads to the increasement of river discharge; at the same time, surface evaporation and plant transpiration becomes heavier, which leads to the reduction in river discharge. In terms of runoff at the Lhasa River, it had decreased since 2004. Therefore, climate change could be affecting the hydrological processes in the Lhasa River Basin [27].

The Lhasa River riparian status was relatively constant, but the submerged area in the midstream had increased in size, while it had reduced in the upstream. The river connection was mainly harmed by the construction of the Pangduo and Zhikong hydropower stations, which have also resulted in the destruction of fish habitat. Prior to the 1990s, the Lhasa River’s fish species were well protected. As a result of large-scale fishing, the fish stocks had sharply declined. Chen and Chen [48] conducted detailed research on the fish found in the upper, middle, and lower reaches of the Lhasa River. They discovered a total of 24 fish species, including 14 indigenous species and 10 exotic species. The primary fish problems were identified as the decline or extinction of indigenous species, such as *Schizopygopsis younghusbandi* and *Glyptosternon maculatum*, and the increase in exotic ones, such as *Crucian*, *Pseudorasbora parva*, and *Loach*. Over-fishing and habitat destruction had resulted in the smaller numbers of native fish found in the basin [48]. For instance, *Glyptosternon maculatum* was heavily fished in the 1990s and was reported to be nearly extinct in the Lhasa River. On the other hand, exotic fish species were introduced via release and were primarily found downstream [39].

Furthermore, the water quality on the main stream of the Lhasa River was found to be in a good condition. The results placed it within the grade Ⅱ limit set by the Environmental Quality Standard of Surface Water [41]. However, ammonia nitrogen and cadmium concentrations occasionally exceed the recommended limits. The increased ammonia nitrogen concentration in wetland during the wet period could be closely related to the hydrological processes of the wetland, as well as to the human activities in the Lhasa River Basin. Numerous wetlands are located on both sides of the river, and their flat terrain and high biomass make them an ideal location for establishing animal husbandry practices [49]. Due to the reduced surface runoff during droughts and the disconnection of rivers and wetlands, ammonia nitrogen in wetlands cannot affect the quality of riverine water. On the other hand, during the wet season, rainwater and snowmelt transport ammonia nitrogen from wetlands to rivers, increasing ammonia nitrogen concentration. Additionally, accelerated litter and humus decomposition in wetlands are potentially important factors affecting riverine water quality [50]. The cadmium concentration exceeded the recommended limit in the year of 2011, which may be associated with the exploitation of mineral resources and hydrological processes. The river basin contains several metal mining sites. Pollution from mining and washing can enter the Lhasa River via rainwater or waste discharge. Due to the low riverine discharge during drought periods, the pollutant concentration increased.

### 4.2. Social Functions

Water resources of the Lhasa River Basin totaled 115.4 × 10^9^ m^3^, with an average annual water supply of 5.2 × 10^9^ m^3^. Except for the Chengguan District in the lower reaches of the Lhasa River Basin, where the utilization rate exceeded 15%, the basin’s overall water resource utilization rate was less than 5%, meeting the needs of water demand of the basin. Furthermore, dykes mostly met the flood-control requirements. The river segments that did not meet the flood control standards and must be strengthened or rebuilt were mainly concentrated primarily in the lower reaches. The construction and operation of Pangduo water conservancy project had significantly increased the irrigation area, as well as the hydropower development rate. The Pangduo irrigated Meda, Lhunzhub, and Chushur irrigation districts, which cover a combined area of more than 2.5 × 10^4^ hm^2^ [29]. In accordance with the Comprehensive Planning of the Lhasa River Basin in 2009, after the construction of Pangduo, runoff during drought periods had increased, and the guaranteed rate of industrial water use in Lhasa also increased from 58.3% to 91.7%. Additionally, the hydropower station generated an average of 5.39 × 10^8^ kWh of electricity per year, greatly accelerating the development of hydropower in the Lhasa River Basin. Landscape planning began in 2012 with the goal of improving the landscape downstream of the Lhasa River. A water retention project had been constructed to create landscape water bodies, and hills on both sides of the river had been reforested to enhance the environment.

In this study, there were six indicators (C6, C7, C8, C12, C14, and C16) in the excellent state, four indicators (C4, C5, C10, and C11) in the good state, three indicators (C3, C9, and C15) in the medium state, and three indicators (C1, C2, and C13) in the poor state. The health level of each indicator in the Lhasa River was similar to the result by Zhang et al. [16]. Additionally, the coordinated development degree model can reflect the synergy and harmony between the natural and social functions of a riverine ecosystem. In a nutshell, riverine health assessment is critical for the sustainable use of water resources. Although a series of measures (e.g., ecological restoration of typical rivers and the implementation of river chief system) had been designed recently to enhance the level of riverine health in China [51], river management continues to face challenges due to urbanization and hydraulic project construction in the Lhasa River Basin. Therefore, it is necessary to strengthen the monitoring of both water quantity and quality, physical habitat, and aquatic life to mitigate the negative influences on riverine health. Moreover, further clarification is required on the impact of hydraulic project construction and operation on riverine ecosystems, as well as on the optimization of the reservoir dispatching process.

## 5. Conclusions

To conduct a thorough assessment of the health state of the Lhasa River in China, an indicator system incorporating both natural and social functions was established. The entropy weight method is used to calculate the weight of riverine health indicators. Subsequently, a model of coordinated development degree was developed to assess riverine health. The results presented that scores for several indicators of natural function, such as flow deviation, ecological flow satisfaction, and riparian status, decreased with the increasing human interference. The scores for water resource utilization, water supply, and hydropower development were increasing, indicating that the Lhasa River performed an increasing number of social functions for humans. The riverine state index of the Lhasa River was ranked as good, with a gradually increasing trend from 0.639 to 0.671. Additionally, it decreased from upstream to downstream. Throughout the study period, the coordinated development degree developed from “moderately” to “highly”, with the values of 0.799, 0.805, 0.819, and 0.817, respectively. In the year of 2011 and 2012, social function scored lower than natural function, but then exceeded natural function in 2013 and 2014.

The coordinated development degree model is characterized by simplicity, generality, and comprehensiveness. The results can be used to assess not only the health of individual river sections and the entire river, but also the coordinated development of natural and social attributes for characterizing the Lhasa River. It is important to note that the riverine state index and coordinated development degree can provide scientific support for riverine system management, ecological restoration, and ecosystem service conservation. This paper proposes the assessment of riverine health using the coordinated development degree model, which still requires improvements in the future. First, to balance the impact of subjective willingness and objective data on the result, the subjective weight method and the entropy weight method can be combined to enhance the rationality of the comprehensive evaluation result. Finally, the natural and socioeconomic background of each monitoring station vary greatly for large rivers. Therefore, the targeted riverine health assessment should be performed in accordance with the demands of different river sections.

## Figures and Tables

**Table 1:** The list of indicators for riverine health assessments.

Target Layer A		Criterion Layer B	Index Layer C	Data Source
**A1 Riverine health**	Natural functions	B1 Hydrology	C1 Flow deviation	Hydrological monitoring data
			C2 Ecological flow satisfaction	
		B2 Physical form	C3 Riparian status	Remote sensing and field investigation
			C4 River connectivity	
			C5 Wetland retention	Water conservancy departments
		B3 Water quality	C6 DO concentration	Tibet Autonomous Region Water Environment Monitoring Center
			C7 Nutrient concentration	
			C8 Heavy metal pollutants	
		B4 Aquatic life	C9 Biodiversity	Field survey
			C10 Fish	
	Social functions	B5 Flood control function	C11 Flood control	Water conservancy departments
		B6 Loading pollutant function	C12 Water function zone	Tibet Water Resources Bulletin
		B7 Water supply function	C13 Water resource utilization	Tibet Water Resources Bulletin
			C14 Water supply	Tibet Water Resources Bulletin
			C15 Hydropower development	Water conservancy departments
		B8 Landscape function	C16 Public satisfaction	Questionnaire

**Table 2:** The scoring criteria for riverine health indicators.

Index	Scores							Multi-Point Score Rule
	1.0	0.8	0.6	0.4	0.3	0.2	0	
C1	0.05	0.1	0.3	0.8		2.0	5.0	—
C2	*EF*_1_ = 50% ^a^ *EF*_2_ = 30%	*EF*_1_ = 40% *EF*_2_ = 20%	—	*EF*_1_ = 30% *EF*_2_ = 10%	—	*EF*_1_ = 10% *EF*_2_ = 5%	*EF*_1_ < 10% *EF*_2_ < 5%	—
C3	Specific evaluation standard can be seen in the Ministry of Water Resources [42].							—
C4	0	—	0.25	0.5	—	0.2	0	—
C5	95%	—	90%	—	80%	75%	60%	—
C6	7.5	6.0	5.0	—	3.0	—	0	The lowest scores from C6 to C8 were taken as the evaluation result of the entire river reach [41].
C7	*CODMN_r_* = 2 ^b^ *COD_r_* = 15 *BOD_r_* = 3 *NH_3_–N_r_* = 0.15	*CODMN_r_* = 4 *COD_r_* = 17.5 *BOD_r_* = 3.5 *NH_3_–N_r_* = 0.5	*CODMN_r_* = 6 *COD_r_* = 20 *BOD_r_* = 4 *NH_3_–N_r_* = 1	—	*CODMN_r_* = 10 *COD_r_* = 30 *BOD_r_* = 6 *NH_3_–N_r_* = 1.5	—	*CODMN_r_* = 15 *COD_r_* = 40 *BOD_r_* = 10 *NH_3_–N_r_* = 2	
C8	*Hg_r_* = 0.00005 *Cd_r_* = 0.001 *Cr_r_* = 0.01 *Pb_r_* = 0.01 *As_r_* = 0.05	—	*Hg_r_* = 0.0001 *Cd_r_* = 0.005 *Cr_r_* = 0.05 *Pb_r_* = 0.05 *As_r_* = 0.075	—	—	—	*Hg_r_* = 0.001 *Cd_r_* = 0.01 *Cr_r_* = 0.1 *Pb_r_* = 0.1 *As_r_* = 0.1	
C9	4	3.2	2.4	1.6	1.2	0.8	0	The average score of C9 and C10 were taken as the evaluation result of the entire river reach.
C10	1.0	0.85	0.75	0.6	0.5	—	0	
C11	95%	90%	85%	80%	—	70%	50%	—
C12	100%	80%	60%	40%	30%	20%	0	—
C13	A value of C13 that is too high or too low does not meet the requirements for riverine health assessment. It is internationally recognized that a reasonable water resource utility rate should be between 30% and 40%. Even if all of the available rainwater and flood resources are to be fully utilized, the rate should not exceed 60%. The relationship between C13 and its score is as follows: $WRU_{s}=a\times {\left(WRU\right)}^{2}+b\times \left(WRU\right)$, where *WRU_s_* is the parabolic distribution of C13; and the values of *a* and *b* are 1111.11 and 666.67, respectively.							—
C14	100%	80%	60%	40%	30%	20%	0	—
C15	10%	20%	30%	40%	—	50%	≥60%, 0	—
C16	The average score of C16 is obtained based on the public participation survey statistics.							—

^a^ *EF*_1_ and *EF*_2_ represent the environmental flow guarantee degree in spawning period (from April to September) and normal period (from October to March); ^b^ *CODMN_r_*, *COD_r_*, *BOD_r_*, and *NH_3_*–*N_r_* represent the concentration of permanganate index, chemical oxygen demand, five-day biochemical oxygen demand, and ammonia nitrogen, respectively.

**Table 3:** The evaluation criteria and classification standards for the riverine state index, coordinated development degree, and relative development degree.

Riverine State Index, *T* [44]		Coordinated Development Degree, *D* [44]		Relative Development Degree, *E*	
0–0.2	Critical	0–0.2	Severely uncoordinated development	<0.8	Slow development of the natural functions
0.2–0.4	Poor	0.2–0.4	Moderately uncoordinated development		
0.4–0.6	Medium	0.4–0.6	Barely coordinated development	0.8–1.2	Synchronized development
0.6–0.8	Good	0.6–0.8	Moderately coordinated development	>1.2	Slow development of the social functions
0.8–1.0	Excellent	0.8–1.0	Highly coordinated development		

## Data Availability

Not applicable.

## Supplementary Materials

The following supporting information can be downloaded at: https://www.mdpi.com/article/10.3390/ijerph19127182/s1. Table S1: Index description and calculation methods of Lhasa River health assessments; Table S2: Index weights of the Lhasa River health assessment.

## References

[1] Grizzetti B., Lanzanova D., Liquete C., Reynaud A., Cardoso A.C. (2016). Assessing water ecosystem services for water resource management. Environ. Sci. Policy.

[2] Intralawan A., Wood D., Frankel R., Costanza R., Kubiszewski I. (2018). Tradeoff analysis between electricity generation and ecosystem services in the Lower Mekong Basin. Ecosyst. Serv..

[3] Bangash R.F., Passuello A., Sanchez-Canales M., Terrado M., López A., Elorza F.J., Ziv G., Acuña V., Schuhmacher M. (2013). Ecosystem services in Mediterranean river basin: Climate change impact on water provisioning and erosion control. Sci. Total Environ..

[4] Wang L., Wang Y., Li Y., Zhang W., Zhang H., Niu L., Habibul N. (2022). Benthic Biofilm Bacterial Communities and Their Linkage with Water-Soluble Organic Matter in Effluent Receivers. Int. J. Environ. Res. Public Health.

[5] Chen J., Mei Y., Ben Y., Hu T. (2020). Emergy-based sustainability evaluation of two hydropower projects on the Tibetan Plateau. Ecol. Eng..

[6] Zuo Q., Zhao H., Mao C., Ma J., Cui G. (2015). Quantitative analysis of human-water relationships and harmony-based regulation in the Tarim River Basin. J. Hydrol. Eng..

[7] Singh P.K., Saxena S. (2018). Towards developing a river health index. Ecol. Indicat..

[8] Scrimgeour G.J., Wicklum D. (1996). Aquatic ecosystem health and integrity: Problems and potential solutions. J. N. Am. Benthol. Soc..

[9] Karr J.R. (1999). Defining and measuring river health. Freshw. Biol..

[10] Gao X., Zhao S., Zhang C., Tu X. (2009). Index system and method for assessing the health status of river. J. Hydraul. Eng..

[11] Luo Z., Zuo Q., Shao Q. (2018). A new framework for assessing river ecosystem health with consideration of human service demand. Sci. Total Environ..

[12] Wang S., Zhang Q., Yang T., Zhang L., Li X., Chen J. (2019). River health assessment: Proposing a comprehensive model based on physical habitat, chemical condition and biotic structure. Ecol. Indicat..

[13] Wu J., Mao R., Li M., Xia J., Song J., Cheng D., Sun H. (2020). Assessment of aquatic ecological health based on determination of biological community variability of fish and macroinvertebrates in the Weihe River Basin, China. J. Environ. Manag..

[14] Vollmer D., Shaad K., Souter N.J., Farrell T., Dudgeon D., Sullivan C.A., Fauconnier I., MacDonald G.M., McCartney M.P., Power A.G. (2018). Integrating the social, hydrological and ecological dimensions of freshwater health: The Freshwater Health Index. Sci. Total Environ..

[15] Zhang X., Meng Y., Xia J., Wu B., She D. (2018). A combined model for river health evaluation based upon the physical, chemical, and biological elements. Ecol. Indicat..

[16] Zhang Z., Li Y., Wang X., Li H., Zheng F., Liao Y., Tang N., Chen G., Yang C. (2021). Assessment of river health based on a novel multidimensional similarity cloud model in the Lhasa River, Qinghai-Tibet Plateau. J. Hydrol..

[17] Cui D., Chen X., Xue Y., Li R., Zeng W. (2019). An integrated approach to investigate the relationship of coupling coordination between social economy and water environment on urban scale—A case study of Kunming. J. Environ. Manag..

[18] Xu W., Dong Z., Ren L., Ren J., Guan X., Zhong D. (2019). Using an improved interval technique for order preference by similarity to ideal solution to assess river ecosystem health. J. Hydroinformatics.

[19] Deng X., Xu Y., Han L., Yu Z., Yang M., Pan G. (2015). Assessment of river health based on an improved entropy-based fuzzy matter-element model in the Taihu Plain, China. Ecol. Indicat..

[20] Safavi H.R., Ahmadi K.M. (2015). Prediction and assessment of drought effects on surface water quality using artificial neural networks: Case study of Zayandehrud River, Iran. J. Environ. Health Sci..

[21] Gao Y., Wu Z., Lou Q., Huang H., Cheng J., Chen Z. (2012). Landscape ecological security assessment based on projection pursuit in Pearl River Delta. Environ. Monit. Assess..

[22] Long Y., Xu G., Ma C., Chen L. (2016). Emergency control system based on the analytical hierarchy process and coordinated development degree model for sudden water pollution accidents in the Middle Route of the South-to-North Water Transfer Project in China. Environ. Sci. Pollut. Res..

[23] Sun Q., Zhang X., Zhang H., Niu H. (2018). Coordinated development of a coupled social economy and resource environment system: A case study in Henan Province, China. Environ. Dev. Sustain..

[24] Munda G. (2005). “Measuring Sustainability”: A Multi-Criterion Framework. Environ. Dev. Sustain..

[25] Wang Q., Yuan X., Cheng X., Mu R., Zuo J. (2014). Coordinated development of energy, economy and environment subsystems—A case study. Ecol. Indicat..

[26] Xing L., Xue M., Hu M. (2019). Dynamic simulation and assessment of the coupling coordination degree of the economy–resource–environment system: Case of Wuhan City in China. J. Environ. Manag..

[27] Chen J., Mei Y., Xiao W. (2019). Establishment of the ecological relationships and properties of the Lhasa River Basin water resources system, China. Sustain. Cities Soc..

[28] Wu Z., Mei Y., Chen J., Hu T., Xiao W. (2019). Attribution analysis of dry season runoff in the Lhasa River using an extended hydrological sensitivity method and a hydrological model. Water.

[29] Li Q., Song J., Wei A., Zhang B. (2013). Changes in major factors affecting the ecosystem health of the Weihe River in Shaanxi Province, China. Front. Env. Sci. Eng..

[30] Tang D., Liu X., Zou X. (2018). An improved method for integrated ecosystem health assessments based on the structure and function of coastal ecosystems: A case study of the Jiangsu coastal area, China. Ecol. Indicat..

[31] Zhao Y.W., Zhou L.Q., Dong B.Q., Dai C. (2019). Health assessment for urban rivers based on the pressure, state and response framework—A case study of the Shiwuli River. Ecol. Indicat..

[32] Brierley G., Reid H., Fryirs K., Trahan N. (2010). What are we monitoring and why? Using geomorphic principles to frame eco-hydrological assessments of river condition. Sci. Total Environ..

[33] Maddock I. (1999). The importance of physical habitat assessment for evaluating river health. Freshw. Biol..

[34] Amiri R., Weng Q., Alimohammadi A., Alavipanah S.K. (2009). Spatial–temporal dynamics of land surface temperature in relation to fractional vegetation cover and land use/cover in the Tabriz urban area, Iran. Remote Sens. Environ..

[35] Nan S., Li J., Zhang L., An R., Pu C., Huang W. (2018). Distribution characteristics of phosphorus in the Yarlung Zangbo River Basin. Water.

[36] Strong W.L. (2016). Biased richness and evenness relationships within Shannon–Wiener index values. Ecol. Indicat..

[37] Zuo Q., Luo Z., Ding X. (2016). Harmonious development between socio-economy and river-lake water systems in Xiangyang City, China. Water.

[38] Qi L., Huang J., Huang Q., Gao J., Wang S., Guo Y. (2018). Assessing aquatic ecological health for Lake Poyang, China: Part I Index development. Water.

[39] Fan L., Liu H., Lin J., Pu Q. (2016). Non-native fishes: Distribution and assemblage structure in the Lhasa River Basin, Tibet, China. Acta Hydrobiol. Sin..

[40] Goss-Custard J.D., Triplet P., Sueur F., West A.D. (2006). Critical thresholds of disturbance by people and raptors in foraging wading birds. Biol. Conserv..

[41] State Environmental Protection Administration (SEPA) (2002). Environmental Quality Standard of Surface Water (GB3838-2002). https://www.mee.gov.cn/ywgz/fgbz/bz/bzwb/shjbh/shjzlbz/200206/t20020601_66497.shtml.

[42] Ministry of Water Resources (2010). River and Lake Health Assessment Guide (trial). http://www.gov.cn/xinwen/2020-08/17/content_5535367.htm.

[43] Pan G., Xu Y., Yu Z., Song S., Zhang Y. (2015). Analysis of river health variation under the background of urbanization based on entropy weight and matter-element model: A case study in Huzhou City in the Yangtze River Delta, China. Environ. Res..

[44] Zhai J., Xu G., Guo S., Wang Y. (2016). Research on river health assessment method based on coordinated development degree. J. Hydraul. Eng..

[45] Yu B., Xu L., Wang X. (2016). Ecological compensation for hydropower resettlement in a reservoir wetland based on welfare change in Tibet, China. Ecol. Eng..

[46] Lin X., Zhang Y., Yao Z., Gong T., Wang H., Chu D., Liu L., Zhang F. (2010). The trend on runoff variations in the Lhasa River Basin. J. Geogr. Sci..

[47] Labat D., Goddéris Y., Probst J.L., Guyot J.L. (2004). Evidence for global runoff increase related to climate warming. Adv. Water Resour..

[48] Chen F., Chen Y. (2010). Investigation and protection strategies of fishes of Lhasa River. Acta Hydrob Sin..

[49] Zhang Y., Wang C., Bai W., Wang Z., Tu Y., Yangjaen D.G. (2008). Alpine wetlands in the Lhasa River Basin, China. J. Geogr. Sci..

[50] Zhai J., Cong L., Yan G., Wu Y., Liu J., Wang Y., Zhang Z., Zhang M. (2019). Influence of fungi and bag mesh size on litter decomposition and water quality. Environ. Sci. Pollut. Res..

[51] Wang Y., Chen X. (2020). River chief system as a collaborative water governance approach in China. Int. J. Water Resour. Dev..

