# Supplementary material for: Riverine Health Assessment Using Coordinated Development Degree Model Based on Natural and Social Functions in the Lhasa River, China
Source: Int J Environ Res Public Health. 2022 Jun 11;19(12):7182. doi: 10.3390/ijerph19127182 (PMC9223359; doi:10.3390/ijerph19127182)
Supplement: Supplementary file 1 [file ijerph-19-07182-s001.zip › ijerph-1718519-supplementary.pdf]

Table S1. Index description and calculation methods of Lhasa River health assessments.

Index name	Index interpretation	Calculation method
C1 Flow deviation	C1 represents the deviation between measured runoff and natural runoff, mainly affected by the development and utilization of basin water resources.	$C1=\sqrt{\sum_{m=1}^{12}\left(\frac{q_m-Q_m}{Q_a}\right)^2}$, $Q_a=\frac{1}{12}\sum_{m=1}^{12}Q_m$, where q_m is the measured monthly runoff, m^3; Q_m is the natural monthly runoff, m^3; Q_a is the annual average natural monthly runoff, m^3.
C2 Ecological flow satisfaction	C2 is the minimum runoff required to maintain the survival and population structure of fish and aquatic plants.	$C2=\min\left(EF_1,EF_2\right)$, $EF_1=\min\left[\frac{q_d}{Q}\right]_{m=4}^{9}$, $EF_2=\min\left[\frac{q_d}{Q}\right]_{m=10}^{3}$, where EF_1 and EF_2 represent environmental discharge guarantee degree in spawning period (from April to September) and normal period (from October to March); q_d is the measured daily runoff, m^3; Q is the average annual runoff, m^3.
C3 Riparian status	C3 is a comprehensive index comprising of bank stability, fractional vegetation cover and riparian disturbance.	Riverbank stability is related to slope inclination, altitude, material, and erosion intensity. Fractional vegetation cover refers to the percentage of the vertical projection area of vegetation on the ground to the total statistical area. Riparian disturbance refers to the negative impact of human activities on riparian, including sand mining, coastal buildings, railways, landfills, pipelines, cultivation, and livestock breeding.
C4 River connectivity	C4 mainly evaluates the blockage of fish migration, water flow, and nutrient transfer.	C4 is obtained according to the number of buildings or facilities affecting river connectivity per unit river length (100 km).
C5 Wetland retention	C5 is the ratio of natural wetland area in assessment year to historical wetland area. This	$C5=AWA/HWA$, where AWA is natural wetland area in assessment year, km^2; and HWA is natural wetland area in 1950, km^2.

Index name	Index interpretation	Calculation method
	paper selects 1950 as the historical year, which before exclusive urbanization development.	
C6 DO concentration	C6 refers to dissolved oxygen concentration in water.	$C6 = DO_r$, where DO_r indicates dissolved oxygen concentration.
C7 Nutrient concentration	C7 is the score of organic pollutants including permanganate index (COD_{Mn}), chemical oxygen demand (COD), five-day biochemical oxygen demand (BOD_5), and ammonia nitrogen (NH_3-N).	$C7 = \min\left(CODMN_r, COD_r, BOD_r, NH3N_r\right)$, where $CODMN_r$, COD_r, BOD_r and $NH3N_r$ are the concentration of COD_{Mn}, COD, BOD_5, and NH_3-N, respectively.
C8 Heavy metal pollutants	C8 refers to the water pollution by heavy metal pollutants and compounds with biological toxicity, such as mercury (Hg), cadmium (Cd), chromium (Cr), lead (Pb) and arsenic (As).	$C8 = \min\left(Hg_r, Cd_r, Cr_r, Pb_r, As_r\right)$, where Hg_r, Cd_r, Cr_r, Pb_r, and As_r are the concentration of Hg, Cd, Cr, Pb, and As, respectively.
C9 Biodiversity	C9 evaluates environmental quality using Shannon-wiener biodiversity index.	$C9 = -\sum_{i=1}^{S}\left(n_i/N\right)\log_2\left(n_i/N\right)$, where S is species numbers; n_i is the number of individuals in one species; N is the total number of individuals.
C10 Fish	C10 indicates the losses of fish in river ecosystems.	$C10 = FA/FR$, where FA and FR are the fish populations in assessment year and reference situation.
C11 Flood control	C11 indicates the current situation of flood storage area, dikes and river reconstruction in the river basin.	$C11 = RLS/RL$, where RLS is the length of river reach that meets the flood control requirements; RL is the total length of river reach.

Index name	Index interpretation	Calculation method
C12 Water function zone	C12 indicates the current situation of water quality in watershed water function regions.	$C12 = NRS/NR$, where NRS is the number of water function regions that water quality attaining standard; NR is the total number of water function regions.
C13 Water resource utilization	C13 expresses the influence of social activities on water quantity, reveals the coordination between socio-economic development and environmental protection.	$C13 = WU/WR$, where WU and WR are the water supply and total water resources, respectively.
C14 Water supply	C14 is the ratio of total water supply to total water demand, which reflects the satisfaction degree of rivers to the water demand for the development of domestic economy in the surrounding areas.	$C14 = TS/TD$, where TS and TD are the total water supply and water demand, respectively.
C15 Hydropower development	C15 refers to the ratio of developed installed capacity and potential hydropower resources.	$C15 = IC/PH$, where IC and PH are the developed installed capacity and potential hydropower resources, respectively.
C16 Public satisfaction	C16 refers to the public satisfaction with the assessment of river landscape and aesthetic value.	C16 is obtained based on public participation survey statistics.

Table S2. Index weights of the Lhasa River health assessment.

Target layer		Criterion layer	Weight	Index layer
Riverine health	Natural functions	Hydrology	**0.147**	Flow deviation **(0.054)**
				Ecological flow satisfaction **(0.093)**
		Physical form	**0.164**	Riparian status **(0.056)**
				River connectivity **(0.057)**
				Wetland retention **(0.051)**
		Water quality	**0.161**	DO concentration **(0.053)**
				Nutrient concentration **(0.057)**
				Heavy metal pollutants **(0.051)**
		Aquatic life	**0.195**	Biodiversity **(0.120)**
				Fish losses **(0.075)**
	Social functions	Flood control function	**0.063**	Flood control **(0.063)**
		Loading pollutant function	**0.051**	Water function zone **(0.051)**
		Water supply function	**0.163**	Water resources utilization **(0.058)**
				Water supply guarantee **(0.052)**
				Hydropower development **(0.052)**
		Landscape function	**0.057**	Public satisfaction **(0.057)**
